# Elevated circulating fasting glucagon-like peptide-1 in surgical patients with aortic valve disease and diabetes

**DOI:** 10.1186/s13098-017-0279-0

**Published:** 2017-10-10

**Authors:** Camilla Krizhanovskii, Stelia Ntika, Christian Olsson, Per Eriksson, Anders Franco-Cereceda

**Affiliations:** 1 Department of Internal Medicine, Södertälje Hospital, 152 86 Södertälje, Sweden; 2 Department of Molecular Medicine and Surgery, Karolinska Institute, Stockholm, Sweden; 3 Cardiovascular Medicine Unit, Department of Medicine, Karolinska Institutet, Karolinska University Hospital Solna, Stockholm, Sweden

**Keywords:** Aneurysm, Diabetes, Aortic valve disease, Glucagon-like peptide-1

## Abstract

**Background:**

Diabetes is a risk factor for peripheral, coronary, and cerebrovascular disease. In contrast, results also indicate that patients with diabetes have reduced prevalence of aortic aneurysms, although the mechanisms remain largely unknown. We hypothesize that altered endogenous secretion of the intestinal hormone glucagon-like peptide-1 (GLP-1)—previously shown to protect from aneurysm formation, and governing many of the mechanisms thought to be involved in aneurysm formation—may provide insights into the mechanisms underlying the inverse relationship of diabetes and aneurysm.

**Methods:**

We undertook a case–control study to characterize circulating plasma GLP-1 levels in diabetic and non-diabetic surgical patients with aortic valve disease, and with or without ascending aortic dilation. The cohort included patients with a bicuspid aortic valve (BAV), a common congenital disorder associated with ascending aortic aneurysm, as well as patients with a tricuspid aortic valve (TAV).

**Results:**

In our patient group, diabetes was characterized by a significant increase in fasting plasma GLP-1 levels. Further, we show that aortic dilation in these patients was associated with a significant increase in fasting plasma GLP-1, although a significant increase in the intact and bioactive peptide could not be detected in BAV patients with aortic dilation.

**Conclusion:**

A subgroup of diabetic patients with aortic valve pathology have increased fasting plasma GLP-1 levels, which may be of importance for the low prevalence of aortic dilation in this patient group. Further, in TAV patients, GLP-1 secretion and plasma levels of intact GLP-1 are upregulated in association with aortic dilation, possibly indicating a compensatory mechanism.

**Electronic supplementary material:**

The online version of this article (doi:10.1186/s13098-017-0279-0) contains supplementary material, which is available to authorized users.

## Background

Diabetes is an established risk factor for peripheral, coronary, and cerebrovascular disease. However, results demonstrate that patients with diabetes have reduced prevalence of thoracic aortic aneurysms (TAA) and abdominal aortic aneurysms (AAA) [1, 2]. In addition, the decreased rate of hospitalization due to thoracic aortic aneurysms and dissections (TAAD) is proportional to the severity of diabetic complications [3].

The mechanisms underlying the protective effect of diabetes are suggested to involve clinical factors including improved blood pressure control and pharmacological treatment, as well as molecular mechanisms involving cross-linking of collagen in the aortic wall and altered proteolytic activity. Aneurysm in the ascending aorta is a common complication in patients born with a bicuspid (BAV) instead of a tricuspid (TAV) aortic valve. BAV is the most common cardiac anomaly and present in 1–2% of the population. BAV patients have an increased prevalence of aortic stenosis (AS), and significantly higher risk of developing TAA than individuals with TAV [4].

Previous studies indicate that the mechanisms underlying the formation of aneurysm may be different in BAV and TAV patients [5].

The intestinal hormone glucagon-like peptide-1 (GLP-1) may provide clues to some of the mechanisms underlying the inverse relationship of diabetes and aneurysm. GLP-1 is a peptide hormone with a central role in type 2 diabetes (T2D), exerting a wide range of effects on glucose metabolism and cardiovascular function [6]. The release of GLP-1—synthesized from the preproglucagon gene (*Gcg*) in a subset of enteroendocrine cells (EECs), denominated L-cells—is stimulated by nutrient intake and potentiates glucose-stimulated insulin secretion (GSIS). In the circulation, the majority of biologically active GLP-1 is found in the GLP-1(7-36) amide form, but also as bioactive GLP-1(7-37). Reduced GLP-1 plasma levels, likely as a consequence of the metabolic state, have been observed in T2D, and with increased BMI/obesity independent of T2D [7, 8]. However, possible over and under-production of GLP-1 remains controversial and probably depends on the severity of the disease [9, 10]. GLP-1 is rapidly degraded mainly by dipeptidyl peptidase-4 [DPP-4] resulting in the metabolite GLP-1 (9-36) and only approx. 10–15% of intact GLP-1 reaching the circulation, and fasting concentrations of bioactive GLP-1 are very low with reported concentrations ranging from 0 to 15 pmol/l [11]. Continuous administration of GLP-1 to T2D patients restores GSIS and normalizes glycemia [12], and stable analogs of GLP-1 (avoiding rapid degradation by DPP-4] are currently among the best available treatments of T2D. Further, the first-line treatment for T2D, metformin, has been indicated to confer some of its effects through increased GLP-1 secretion [13–16].

Whereas the postprandial release and bioactive GLP-1 governs the enhanced GSIS, fasting serum GLP-1 levels have been implicated in playing a role in the regulation of glucagon secretion, energy expenditure, satiety and cardiovascular effects, where GLP-1 metabolites (GLP-1 9-36) are implicated in mediating some of the cardiovascular effects of GLP-1 [17, 18]. In addition, GLP-1 was recently shown to prevent the formation of AAA [19] and the well-known antioxidant properties of GLP-1 [20] may be particularly important in aortic media of BAV patients indicated to have an increased susceptibility to reactive oxygen species (ROS) [21]. In addition to its antioxidant and anti-inflammatory effects, GLP-1 also counteracts angiotensin II and affects collagen composition and MMP secretion, factors indicated to be of importance for development of aortic dilation, and indicated to be mediated also by the inactive fragments of the peptide [22, 23].

In the light of altered plasma GLP-1 in diabetes, the reduced prevalence of aneurysm in diabetic patient groups, and indicated protective effects of GLP-1 analogues in models of aortic aneurysm, plasma GLP-1 may not only be identified as a risk factor for aneurysm formation in high-risk patients, but stable GLP-1 analogues currently used in diabetes therapy may be indicated for patients with increased risk of aortic aneurysm. Consequently, the aim of the present study was to characterize GLP-1 plasma levels in patients with disease of the aortic valve and ascending aorta.

## Methods

Fasting plasma GLP-1 concentration was analyzed in patients subjected to aortic valve and/or ascending aortic surgery. Ethical permission was received from the Stockholm Regional Ethical Committee: (Dnr: 2006/784-31/1; approved: 2006-09-15) and (Dnr: 2012/1633-31/4; approved 2012-10-24).

Bicuspid aortic valve and TAV patients were included according to the ASAP protocol [5] and subjected to either valve and/or ascending aortic surgery. Ascending aortic measurements were obtained through intraoperative echocardiography and the aorta was classified as normal or dilated if < 40 or > 45 mm in maximal diameter, respectively. Exclusion criteria were Marfan patients, monocuspid valves and patients with coronary artery disease.

To aid the interpretation of data, patients with coronary artery disease were also excluded from the present study. In total 180 patients, comprising 55 women and 125 men, were included in the study—42 subjects with T2D (13 BAV, 29 TAV) and 138 (81 BAV, 57 TAV) non-diabetics. Sixty of the patients included had aneurysmal enlargement of the aorta—40 with BAV and 20 with TAV (Additional file 1: Table S1). The diagnosis of diabetes was confirmed based on the criteria of the American Diabetes Association (ADA) (2014). Mean age of the patients included was 65 ± 1 years. Fasting (at least 8 h) levels of total GLP-1 were measured (F-GLP-1). In a subset of patient samples, a DPP-4 inhibitor was added immediately after sampling (10 µl/ml, Cat. No.: DPP4, Millipore), and total as well as active GLP-1 levels were measured. The levels of plasma intact (active) GLP-1 (7-36) were measured by an ELISA kit, Cat. No.: EZGLPHS-35K, Millipore. The levels of total GLP-1 (7-36) and GLP-1 (9-36) were measured by an ELISA kit, Cat. No.: EZGLP1T-36K, Millipore.

### Statistical analysis

All two-sample comparisons reported were performed using an unpaired Student *t* test, with the assumption of unequal variance. To evaluate the correlations between GLP-1 and selected variables, we calculated Spearman correlation coefficients between circulating levels of fasting GLP-1 and the relevant variables.

## Results

### In patients with disease of the aortic valve, diabetes is associated with increased fasting plasma GLP-1 levels regardless of anti-diabetic therapy and valve phenotype

**Fig. 1:**
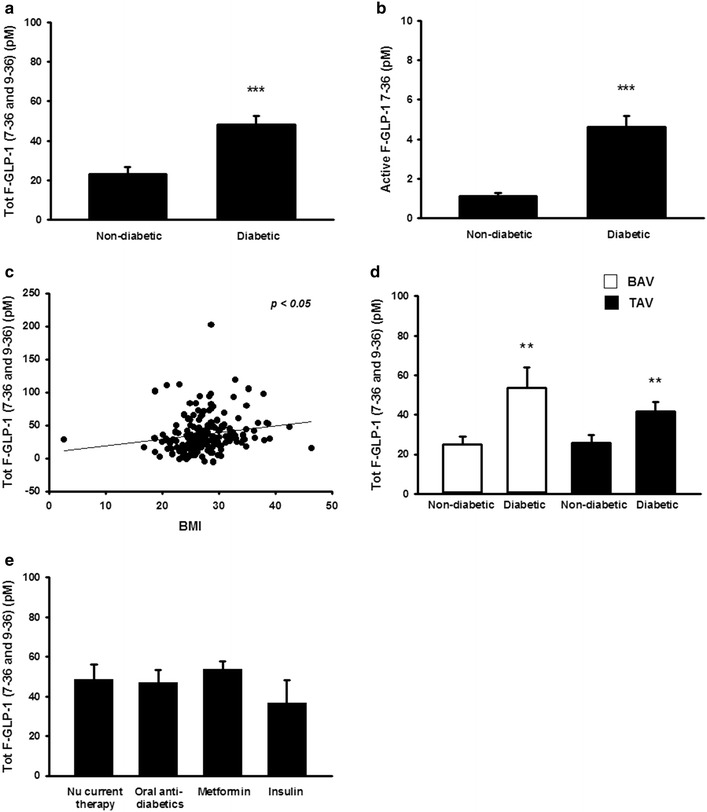
Diabetes is associated with increased fasting plasma GLP-1 levels in patients with disease of the aortic valve. Diabetic subjects had significantly higher levels of fasting plasma total and active GLP-1 as compared to non-diabetic subjects (**a**, **b**), n = 30–45 for individual groups, and a significant positive correlation between BMI and fasting plasma GLP-1 was detected (**c**). Increased fasting plasma levels of total GLP-1 in diabetic patients was observed in BAV as well as TAV patients, with no significant difference in the average GLP-1 plasma level between BAV and TAV patients within the two groups (**d**), n = 12–30 for individual groups. Fasting plasma GLP-1 was not significantly different in diabetic patients receiving pharmacological treatment with anti-diabetics (**e**), n = 6–13 for individual groups. *p < 0.05; **p < 0.01; ***p < 0.001 compared with non-diabetic subjects. Comparisons between groups were made using an unpaired t-test/one-way ANOVA, and Student-Newman-Keul’s post hoc test. Significant correlations were assessed using the Pearson correlation coefficient

The detected concentrations of intact biologically active GLP-1 in plasma for this patient group fall within the expected range and what has previously reported (non-diabetic; 1.3 ± 0.2 pmol/l, diabetic; 4.6 ± 0.6 pmol/l). Further, the total concentrations of GLP-1 reported (non-diabetic; 23.3 ± 3.2 pmol/l, diabetic; 48 ± 4.5 pmol/l) are in agreement with a rapid degradation of the peptide and only approximately 10–15% of intact GLP-1 reaching the circulation A comparison of fasting plasma GLP-1 levels between diabetic and non-diabetic patients with disease of the aortic valve [aortic stenosis or aortic insufficiency (AI)] revealed significantly higher levels of fasting plasma total and active GLP-1 in diabetic subjects as compared to non-diabetic subjects (Fig. 1a, b). Further, a significant positive correlation between BMI and fasting plasma total GLP-1 was detected (Fig. 1c). No significant difference in GLP-1 plasma levels was detected between females and males (data not shown) and the increased plasma levels of GLP-1 in diabetic patients was detected in BAV as well as TAV patients, with no significant difference between the two groups (Fig. 1d). Anti-diabetic drugs such as metformin as well as insulin have been indicated to regulate GLP-1 secretion [13, 14, 16, 24] and may have contributed to the elevated plasma levels of GLP-1 observed in the diabetic patients. To elucidate a possible contribution of pharmacological anti-diabetic treatment to the elevated fasting plasma GLP-1 levels in diabetic patients, we divided the patients into subgroups depending on current medications. However, a comparison of diabetic patients currently receiving oral antidiabetics, metformin monotherapy, insulin monotherapy, or no anti-diabetic medications, revealed no significant differences in total fasting plasma GLP-1 levels (Fig. 1e).

### Increased fasting plasma GLP-1 levels are correlated with increased plasma triglyceride levels, while inversely correlated to fasting plasma glucose and HbA_1c_, in a subgroup of patients with disease of the aortic valve and ascending aorta

**Fig. 2:**
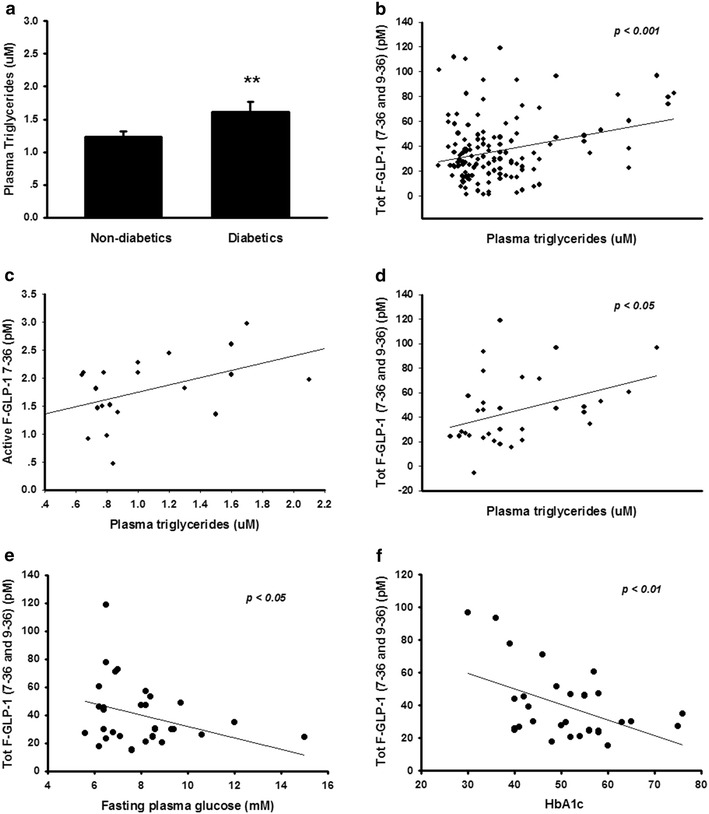
Increased fasting plasma GLP-1 levels are correlated with increased fasting plasma glucose, HbA_1c_ and plasma triglyceride levels. Plasma triglyceride levels were significantly increased in diabetic patients, as compared to non-diabetics (**a**). There was a significant positive correlation between plasma GLP-1 and plasma triglycerides (**b**, **c**); this positive correlation was also detected in diabetic patients (**d**). Fasting plasma GLP-1 showed an inverse relationship to fasting plasma glucose (**e**) and HbA_1c_ (**f**) in diabetic patients. *p < 0.05; **p < 0.01; ***p < 0.001 compared with non-diabetic subjects. Comparisons between groups were made using an unpaired t-test. Significant correlations were assessed using the Pearson correlation coefficient

As reduced plasma fasting and postprandial GLP-1 levels have previously been indicated in diabetes and with increased BMI, we determined how fasting plasma GLP-1 levels in this patient group correlated with the plasma lipid profile and hyperglycemia. Diabetic patients included in this study (fasting plasma glucose and A1C for diabetic vs. non-diabetic; 9 ± 0.7 mmol/l and 47.2 ± 2 mmol/mol vs. 5.5 ± 0.2 mmol/l and 36.9 ± 0.9 mmol/mol respectively) displayed significantly elevated levels of plasma triglycerides (Fig. 2a) as compared to non-diabetic subjects. In determining potential correlations of plasma GLP-1 with lipid profiles and glycemia, we found a significant positive correlation between plasma GLP-1 and plasma triglycerides (Fig. 2b–d). No significant correlation between plasma GLP-1 and total cholesterol, HDL-cholesterol, or LDL-cholesterol could be observed (data not shown). However, there was a significant inverse relationship between fasting plasma glucose and GLP-1 levels, as well as between A1C and GLP-1 levels in the diabetic patients included (Fig. 2e, f).

### Increased fasting plasma GLP-1 levels are observed in patients with aneurysmal enlargement of the aorta

**Fig. 3:**
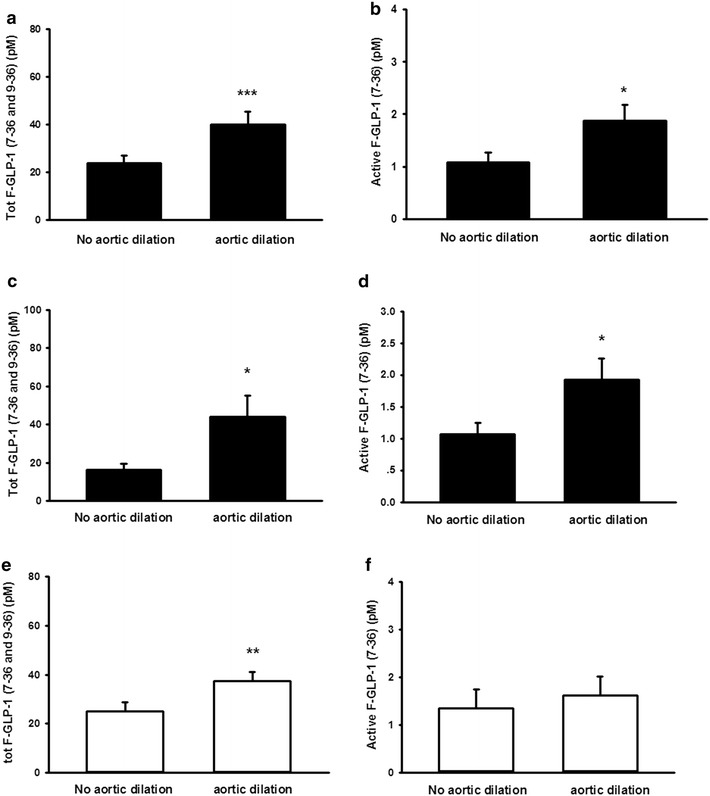
Increased fasting plasma GLP-1 levels are observed in patients with aneurysmal enlargement of the aorta. Fasting plasma total (7-36 and 9-36) (**a**) and active (7-36) GLP-1 (**b**) were significantly increased in patients with aneurysmal enlargement of the aorta. Total (7-36 and 9-36) GLP-1 was significantly increased in TAV as well as BAV patients with aneurysmal enlargement of the aorta (**c**, **e**). This was corroborated by a similar significant increase in fasting plasma active (7-36) GLP-1 in TAV (**d**), but not BAV (**f**), patients with aneurysm. *p < 0.05; **p < 0.01; ***p < 0.001 compared with non-diabetic subjects. Comparisons between groups were made using an unpaired t-test

As we hypothesized that the increased plasma levels of GLP-1 in diabetic subjects may contribute to the low prevalence of aneurysm in this patient group, we analyzed plasma GLP-1 in patients with or without aneurysm, while also determining any potential differences between BAV and TAV patients. Fasting plasma total and active GLP-1 was significantly increased in patients with aneurysmal enlargement of the aorta as compared to patients from the same group without aneurysm (Fig. 3a, b). Dividing the patients into subgroups depending on the aortic valve formation, *i.e.* BAV/TAV, revealed a significant increase in active and total F-GLP-1 levels in TAV patients with aortic dilation (Fig. 3c, d). However, although total GLP-1 levels were also significantly increased in BAV patients (Fig. 3e), the levels of intact active GLP-1 (9-36) were not significantly altered between BAV patients with or without dilation of the aorta (Fig. 3F).

## Discussion

This characterization of GLP-1 plasma levels in patients suffering from disease of the aortic valve and ascending aorta shows a significant increase in fasting plasma GLP-1 in a subgroup of diabetic patients, where the plasma concentrations show a significant positive correlation with the plasma triglyceride levels. Further, non-diabetic BAV and TAV patients with aneurysmal enlargement of the aorta had significantly increased levels of total GLP-1, further reflected by a significant increase also of intact and active GLP-1 in TAV patients. However, no significant increase in plasma intact GLP-1 could be detected in BAV patients.

Reduced plasma levels of GLP-1 have previously been indicated in T2D and linked to impaired glucose tolerance, insulin resistance and increased BMI irrespective of diabetes [7, 8]. In contrast to this, we now report significantly increased levels of plasma GLP-1 in diabetic vs. non-diabetic subjects with disease of the aortic valve and ascending aorta. As the number of patients with AS vs. AI was significantly higher in diabetic patient groups, we did asses GLP-1 plasma levels in AS vs. AI; however, our results demonstrate a trend toward increased levels of fasting plasma GLP-1 in AI vs. AS patients, although no significant difference could be observed (AS plasma FGLP-1; 34.8 ± 3.3, and AI FGLP-1; 49.9 ± 8.9). In addition, a closer look at these diabetic patients revealed that an increase in plasma triglycerides was independently associated with an increase in plasma GLP-1. An increased level of plasma triglycerides is not only a common characteristic of the dyslipidemia associated with insulin resistance and T2D but is also the central pathophysiologic feature of the abnormal lipid profile, previously indicated to perturb GLP-1 secretion [25].

Most studies assessing GLP-1 plasma levels in diabetes and obesity have focused on postprandial levels of bioactive GLP-1. The present study focused on fasting levels of GLP-1, as fasting levels have been indicated to be of importance for the vascular effects of the peptide [18]. Future studies to also characterize postprandial plasma GLP-1 levels for this patient group are underway.

Further, previous studies indicate that the defect in GLP-1 secretion comes as a result of disease progression, likely due to a reduction of GLP-1 secretion [6, 26] following the presence of hyperlipidemia/hyperglycemia and glucolipotoxicity that may directly alter the function and viability of enteroendocrine cells secreting GLP-1 [13, 27]. This may underlie the fact that not all studies have been able to detect reduced plasma GLP-1 levels in diabetic patients, and may also be of importance for the interpretation of the results from the present study. We report herein an inverse relationship between plasma GLP-1 and plasma glucose in diabetic patients, indicating that GLP-1 levels may vary with duration and severity of diabetes. These data are also supported by studies indicating that GLP-1 secretion is not impaired in diabetic patients with well-controlled blood glucose, while diminished in those with poor glycemic control or a longer duration of T2D [10].

Lipids stimulate GLP-1 secretion through enteral mechanisms, as Intralipid i.v. infusion has no effect on circulating levels of GLP-1 [28]. It is possible that an early stage of the disease is characterized by increased circulating levels of GLP-1 due to enhanced GLP-1 secretion in response to dietary fat [28], whereas toxic effects of chronic hyperlipidemia and perhaps a reduced number of enteroendocrine GLP-1 secreting cells at some stage outweigh the stimulatory effect of the enteral fatty acids on GLP-1 secretion. We and others have previously shown increased GLP-1 secretion in response to the anti-diabetic drug metformin [13–15], whereas the present study failed to show a significant increase in fasting GLP-1 levels in patients receiving metformin. However, such a stimulatory effect of metformin cannot be ruled out considering that only fasting levels of GLP-1 were assessed in this study and that a relatively small number of patients receiving metformin monotherapy were included (seven subjects). Our results do, however, indicate that the elevated levels of GLP-1 in diabetic patients with disease of the aortic valve and ascending aorta mainly result from factors other than anti-diabetic pharmaceuticals, as no significant increase was observed in patients receiving oral anti-diabetics, metformin monotherapy or insulin as compared to drug-naïve diabetes patients.

In addition to the well-known anti-inflammatory and vascular effects of GLP-1, a recent study indicates a role for GLP-1 in prevention of abdominal aortic aneurysm in an animal model [19], but it remains to be determined if the increased plasma levels of GLP-1 in the present subgroup of diabetic patients contributes to the reduced prevalence of aneurysm in these patients.

We report herein that fasting GLP-1 levels are significantly increased in patients with disease of the aortic valve and aneurysm, which may suggest that increased endogenous GLP-1 serves as a compensatory mechanism upregulated during the processes contributing to aortic dilation. Possible contributors to elevated GLP-1 levels in patients with aortic dilation could be the presence of inflammation or perhaps diastolic function. However, as the number of diabetic patients with aneurysm was very limited we could not determine any possible differences in GLP-1 plasma levels between diabetic patients with or without aortic dilation. Future larger studies should be conducted to elucidate possible differences in these observed effects between patients with AS/AI.

The present results indicating unaltered levels of the intact GLP-1 peptide in BAV patients with aortic dilation may suggest increased DPP-4 activity in this patient group, which may be of importance for the reported increased sensitivity to reactive oxygen species [21] and formation of aortic aneurysm in BAV patients. However, DPP-4 activity remains to be determined in relevant patient groups, as well as the importance of GLP-1 signaling in the processes that govern aneurysm formation.

## Conclusion

A subgroup of diabetic patients with aortic valve pathology has increased fasting plasma GLP-1 levels, which may be of importance for the low prevalence of aortic dilation in this patient group. However, this increase in GLP-1 plasma levels is likely to be diminished with the duration and severity of diabetes. Further, increased levels of GLP-1 in non-diabetic patients with aortic valve pathology may be an important risk factor for aneurysm formation. Moreover, we conclude that the present report further emphasizes possible differential progression of aneurysm formation in BAV and TAV patients, and may provide an impetus for future clinical studies in which separate analyses are performed for BAV and TAV patients.

## Additional file

**Additional file 1: Table S1.** Patient characteristics.

## Abbreviations

TAA: thoracic aortic aneurysms
AAA: abdominal aortic aneurysms (AAA)
TAAD: thoracic aortic aneurysms and dissections
BAV: bicuspid aortic valve
TAV: tricuspid aortic valve
AS: aortic stenosis
T2D: type 2 diabetes
GLP-1: glucagon-like peptide-1
Gcg: preproglucagon gene
EEC: enteroendocrine cells
GSIS: glucose-stimulated insulin secretion
DPP-4: dipeptidyl peptidase-4
AI: aortic insufficiency
ROS: reactive oxygen species
BMI: body mass index
MMP: matrix metalloproteinase

## References

[1] De Rango P, Farchioni L, Fiorucci B, Lenti M (2014). Diabetes and abdominal aortic aneurysms. Eur J Vasc Endovasc Surg.

[2] Takagi H, Umemoto T, Group A (2017). Negative association of diabetes with thoracic aortic dissection and aneurysm. Angiology.

[3] Jimenez-Trujillo I, Gonzalez-Pascual M, Jimenez-Garcia R (2016). Type 2 diabetes mellitus and thoracic aortic aneurysm and dissection: an observational population-based study in Spain from 2001 to 2012. Medicine (Baltimore).

[4] Losenno KL, Goodman RL, Chu MW (2012). Bicuspid aortic valve disease and ascending aortic aneurysms: gaps in knowledge. Cardiol Res Pract.

[5] Maleki S, Bjorck HM, Paloschi V (2013). Aneurysm Development in patients with bicuspid aortic valve (BAV): possible connection to repair deficiency?. Aorta (Stamford).

[6] Baggio LL, Drucker DJ (2007). Biology of incretins: GLP-1 and GIP. Gastroenterology.

[7] Muscelli E, Mari A, Casolaro A (2008). Separate impact of obesity and glucose tolerance on the incretin effect in normal subjects and type 2 diabetic patients. Diabetes.

[8] Rask E, Olsson T, Soderberg S (2001). Impaired incretin response after a mixed meal is associated with insulin resistance in nondiabetic men. Diabetes Care.

[9] Ahren B, Carr RD, Deacon CF (2010). Incretin hormone secretion over the day. Vitam Horm.

[10] Vollmer K, Holst JJ, Baller B (2008). Predictors of incretin concentrations in subjects with normal, impaired, and diabetic glucose tolerance. Diabetes.

[11] Holst JJ (2007). The physiology of glucagon-like peptide 1. Physiol Rev.

[12] Meier JJ, Nauck MA (2005). Glucagon-like peptide 1(GLP-1) in biology and pathology. Diabetes Metab Res Rev.

[13] Kappe C, Patrone C, Holst JJ, Zhang Q, Sjoholm A (2013). Metformin protects against lipoapoptosis and enhances GLP-1 secretion from GLP-1-producing cells. J Gastroenterol.

[14] Kappe C, Zhang Q, Nystrom T, Sjoholm A (2014). Effects of high-fat diet and the anti-diabetic drug metformin on circulating GLP-1 and the relative number of intestinal L-cells. Diabetol Metab Syndr.

[15] Wu T, Thazhath SS, Bound MJ, Jones KL, Horowitz M, Rayner CK (2014). Mechanism of increase in plasma intact GLP-1 by metformin in type 2 diabetes: stimulation of GLP-1 secretion or reduction in plasma DPP-4 activity?. Diabetes Res Clin Pract.

[16] Mulherin AJ, Oh AH, Kim H, Grieco A, Lauffer LM, Brubaker PL (2011). Mechanisms underlying metformin-induced secretion of glucagon-like peptide-1 from the intestinal L cell. Endocrinology.

[17] Burgmaier M, Liberman A, Mollmann J (2013). Glucagon-like peptide-1 (GLP-1) and its split products GLP-1(9-37) and GLP-1(28-37) stabilize atherosclerotic lesions in apoe(-)/(-) mice. Atherosclerosis.

[18] Matsubara J, Sugiyama S, Sugamura K (2012). A dipeptidyl peptidase-4 inhibitor, des-fluoro-sitagliptin, improves endothelial function and reduces atherosclerotic lesion formation in apolipoprotein E-deficient mice. J Am Coll Cardiol.

[19] Yu J, Morimoto K, Bao W, Yu Z, Okita Y, Okada K (2016). Glucagon-like peptide-1 prevented abdominal aortic aneurysm development in rats. Surg Today.

[20] Batchuluun B, Inoguchi T, Sonoda N (2014). Metformin and liraglutide ameliorate high glucose-induced oxidative stress via inhibition of PKC-NAD(P)H oxidase pathway in human aortic endothelial cells. Atherosclerosis.

[21] Mathieu P, Bosse Y, Huggins GS (2015). The pathology and pathobiology of bicuspid aortic valve: state of the art and novel research perspectives. J Pathol Clin Res.

[22] Robinson E, Tate M, Lockhart S (2016). Metabolically-inactive glucagon-like peptide-1(9-36)amide confers selective protective actions against post-myocardial infarction remodelling. Cardiovasc Diabetol.

[23] Aroor AR, Sowers JR, Jia G, DeMarco VG (2014). Pleiotropic effects of the dipeptidylpeptidase-4 inhibitors on the cardiovascular system. Am J Physiol Heart Circ Physiol.

[24] Thurmond DC (2009). Insulin-regulated glucagon-like peptide-1 release from L cells: actin’ out. Endocrinology.

[25] Ginsberg HN, Zhang YL, Hernandez-Ono A (2005). Regulation of plasma triglycerides in insulin resistance and diabetes. Arch Med Res.

[26] Vilsboll T, Agerso H, Krarup T, Holst JJ (2003). Similar elimination rates of glucagon-like peptide-1 in obese type 2 diabetic patients and healthy subjects. J Clin Endocrinol Metab.

[27] Kuhre RE, Holst JJ, Kappe C (2016). The regulation of function, growth and survival of GLP-1-producing L-cells. Clin Sci (Lond).

[28] Lindgren O, Carr RD, Deacon CF (2011). Incretin hormone and insulin responses to oral versus intravenous lipid administration in humans. J Clin Endocrinol Metab.

