# Supplementary material for: Elevated circulating fasting glucagon-like peptide-1 in surgical patients with aortic valve disease and diabetes
Source: Diabetol Metab Syndr. 2017 Oct 10;9:79. doi: 10.1186/s13098-017-0279-0 (PMC5635503; doi:10.1186/s13098-017-0279-0)
Supplement: Supplementary file 1 — Additional file 1: Table S1. Patient characteristics. [file 13098_2017_279_MOESM1_ESM.docx]

| **Patient group** | **Number of Patients** | **Average Age** | **Gender M/F**  **%** | **BMI** | **Valve pathology AS/AI %** |
| --- | --- | --- | --- | --- | --- |
| **Diabetics** | 42 | 69 | 67/33 | 31* | 95/5 |
| **Non-diabetics** | 138 | 64 | 66/34 | 26 | 71/29 |
| **Diabetics TAV** | 29 | 70 | 76/24 | 31 | 93/7 |
| **Non-diabetics TAV** | 57 | 69 | 63/37 | 27 | 58/42 |
| **Diabetics BAV** | 13 | 67 | 69/31 | 31 | 100/0 |
| **Non-diabetics BAV** | 81 | 62 | 69/31 | 26 | 82/18 |
| **Diabetics no current therapy** | 13 | 68 | 85/15 | 28 | 93/7 |
| **Diabetics oral antidiabetics** | 13 | 69 | 71/29 | 31 | 94/6 |
| **Diabetics Metformin** | 8 | 68 | 57/43 | 32 | 87/13 |
| **Diabetics Insulin** | 7 | 73 | 60/40 | 33 | 100/0 |
| **Non-diabetic aortic dilation** | 60 | 63 | 73/27 | 27 | 65/35 |
| **Non-diabetic no aortic dilation** | 78 | 67 | 61/39 | 26 | 77/23 |
| **Non-diabetic aortic dilation TAV** | 20 | 62 | 67/33 | 29 | 25/75 |
| **Non-diabetic no aortic dilation TAV** | 37 | 73 | 58/42 | 26 | 70/30 |
| **Non-diabetic aortic dilation BAV** | 40 | 63 | 75/25 | 26 | 79/21 |
| **Non-diabetic no aortic dilation BAV** | 41 | 61 | 59/41 | 26 | 79/21 |

*The difference in the median values between the diabetic and ctr groups is greater than would be expected by chance; there is a statistically significant difference (P = <0.001)
